# Osteitis Fibrosa Cystica and pathological fractures—the classic but neglected skeletal manifestation of primary hyperparathyroidism: a case report

**DOI:** 10.1186/s12891-021-04326-1

**Published:** 2021-05-14

**Authors:** Ekasame Vanitcharoenkul, Nontouch Singsampun, Aasis Unnanuntana, Sirinart Sirinvaravong

**Affiliations:** 1 Department of Orthopaedic Surgery, Faculty of Medicine Siriraj Hospital, Mahidol University, Bangkok, Thailand; 2 Division of Endocrinology and Metabolism, Department of Medicine, Faculty of Medicine Siriraj Hospital, Mahidol University, Bangkok, Thailand

**Keywords:** Case report, Osteitis fibrosa cystica, Pathological fracture, Primary hyperparathyroidism, Skeletal manifestation

## Abstract

**Background:**

Osteitis fibrosa cystica is the classic manifestation of primary hyperparathyroidism (PHPT), occurs after prolonged exposure of bone to high serum parathyroid hormone (PTH) level. It has become increasingly rare due to early detection of PHPT.

**Case presentation:**

A 37-year-old woman was referred to our institution for fixation of multiple fractures of upper and lower extremities that had been reoccurring in the past 5 years. Her medical history showed right-shoulder, left-elbow, and right-femur fractures after a fall 5 years previously. One month ago, she sustained fractures of the right distal humerus, left tibia, and left femur without history of trauma. Upon arrival to our hospital, a thorough review of her plain radiographs demonstrated brown tumors at multiple sites, along with a salt-and-pepper appearance of the skull and a rugger-jersey spine, compatible with osteitis fibrosa cystica. Patient was diagnosed with PHPT, confirmed by high-corrected serum calcium (13.6 [8.6–10.0] mg/dl), low serum phosphate (2.2 [2.5–4.5] mg/dL), high serum alkaline phosphatase (1482 [35–105] U/L), and significantly elevated parathyroid hormone (PTH 3850 [15–65] pg/mL). A histologically confirmed, 2.5-cm parathyroid adenoma was removed by parathyroidectomy. Ten days later, closed reduction and internal fixation of the left proximal femoral shaft was performed. Pain and ambulation were significantly improved 6 months postoperatively. At the 1.5-year follow-up, fracture unions and complete mineralization of brown tumors were noted; the patient could ambulate with neither pain nor an assistive device.

**Conclusions:**

PHPT has become more asymptomatic in countries where routine calcium screening is performed. Nevertheless, the classic skeletal involvement, osteitis fibrosa cystica, should not be overlooked, particularly in young patients who present with a low-energy fracture.

**Supplementary Information:**

The online version contains supplementary material available at 10.1186/s12891-021-04326-1.

## Background

Although osteitis fibrosa cystica, or brown tumor, is one of the classic manifestations of primary hyperparathyroidism (PHPT), it is becoming increasingly rare due to early detection of PHPT. Currently, the reported prevalence of osteitis fibrosa cystica in PHPT is less than 2% [1]. We report the case of a 37-year-old woman who had had multiple pathological fractures during the preceding 5 years and whose diagnosis of PHPT was delayed due to a lack of awareness of possible underlying metabolic bone diseases.

## Case presentation

A 37-year-old Thai woman was referred to our institution for fixation of multiple recurring pathological fractures of both upper and lower extremities. Five years ago, she had sustained fractures of the right shoulder, left elbow, and right femoral shaft after falling on the ground. She received internal fixation of those fractures, as illustrated in Supplementary 1. One month previously, she had been admitted to a local hospital because of fractures of the right distal humerus and left tibial shaft without history of trauma. While staying at that hospital, a new fracture occurred at the left femoral shaft. Since complicated orthopedic procedures for fixation of her fractures were anticipated, she was subsequently transferred to our hospital.

She reported urinary frequency, with more than 10 nocturia episodes per night, accompanied by increased thirst. She was a non-smoker and did not have a history of diabetes, hypertension, or other chronic diseases. She denied a family history of any metabolic bone diseases or genetic disorders.

A musculoskeletal examination showed slightly varus malalignment of the right elbow and shortening with external rotation deformity of the left leg, and painful swelling of the left tibia. A neck examination did not reveal thyroid gland enlargement, thyroid nodules, or palpable neck masses.

**Fig. 1:**
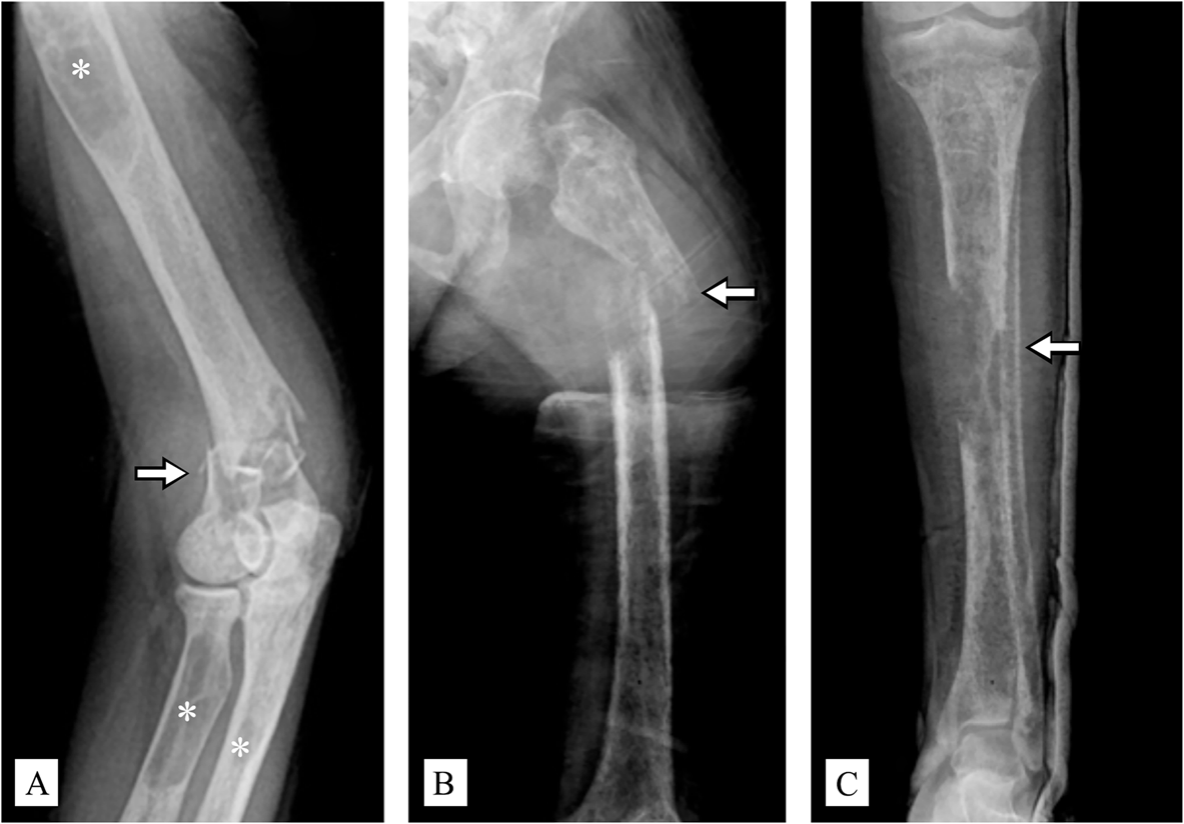
Plain radiographs of the patient. **a** A comminuted fracture across the supracondylar area of the right humerus; and a brown tumor at the shaft of the right humerus, proximal radius, and proximal shaft of the right ulnar. **b** A brown tumor with a pathological fracture at the left proximal femoral shaft, with total displacement. **c** A brown tumor with a pathological fracture at the left tibial shaft (white arrow indicates a pathological fracture; asterisk indicates a brown tumor)

**Fig. 2:**
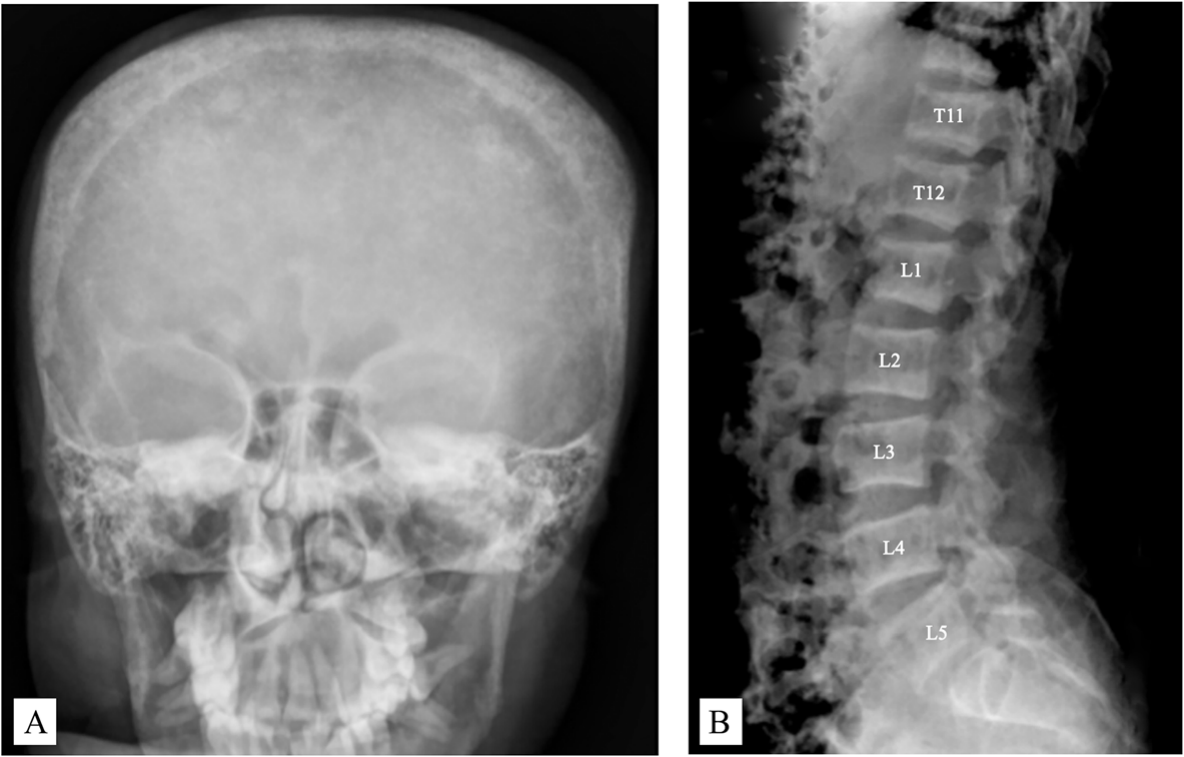
**a** A plain radiograph of the skull showing multiple, tiny, well-defined radiolucent areas on the skullcap, compatible with a salt-and-pepper appearance. **b** A plain radiograph of the spine showing prominent density of the subchondral endplate at multiple lumbar spines, or the rugger-jersey appearance

A review of plain radiographs revealed multiple fractures with diffuse osteopenia. Brown tumors were also observed at multiple sites (the shaft of the right humerus, the proximal shaft of the right radius and ulnar, the proximal shaft of the left femur, and the shaft of the left tibia; Fig. 1). Moreover, a bone survey showed a salt-and-pepper appearance of the skull, while a rugger-jersey appearance was observed in a lateral spine radiograph (Fig. 2). These findings were consistent with osteitis fibrosa cystica, prompting further laboratory investigations for PHPT.

The patient’s initial laboratory investigations are listed in Table 1. She had elevated serum calcium (13.6 mg/dL); low phosphorus (2.2 mg/dL) with high bone turnover, giving markedly elevated alkaline phosphatase (1482 U/L); and very high serum PTH (3850 pg/mL).

**Table 1:**
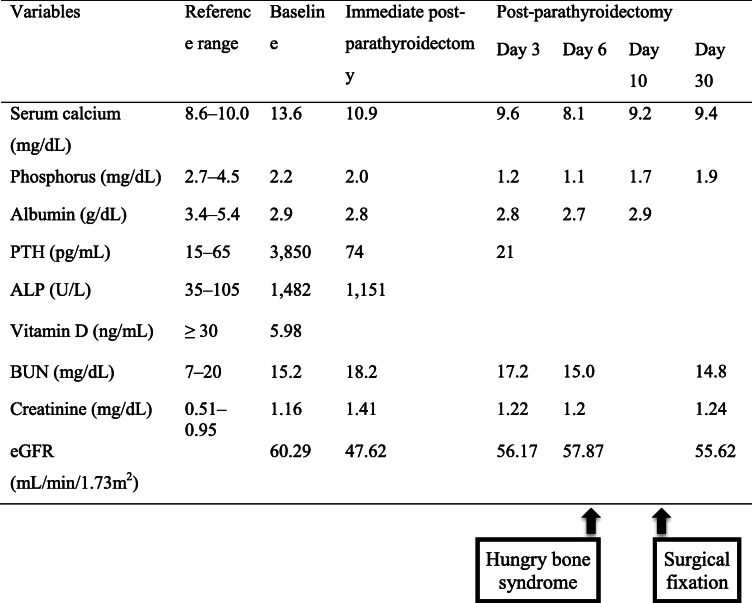
Patient’s laboratory values

Abbreviations: *ALP* Alkaline phosphatase, *baseline* Laboratory results at admission, *BUN* Blood urea nitrogen, *eGFR* Estimated glomerular filtration rate, *post parathyroidectomy* Postoperative parathyroidectomy, *PTH* Parathyroid hormone, *serum calcium* Albumin-corrected serum calcium, *vitamin D* 25-hydroxyvitamin D

The patient was diagnosed with PHPT. Because her serum calcium was high, it was decided that a parathyroidectomy procedure would be performed prior to operative fixation of the fractures. The fractures were temporarily stabilized with a long leg back-slab on the left leg and a U-slab on the left arm. Further investigation with a parathyroid scan (using ^99m^Tc-Sestamibi and single photon emission computed tomography) showed a parathyroid lesion at the inferior pole of the left thyroid lobe. The patient underwent a left lower parathyroidectomy; a 2.5-cm mass that was removed was subsequently histologically confirmed as a parathyroid adenoma.

After removal of the tumor, the patient’s PTH level decreased to 21 pg/mL on postoperative Day 3. Immediately after the surgery, calcium carbonate, alfacalcidol, and vitamin D2 (ergocalciferol) supplementation were prescribed as part of a prophylactic protocol to prevent postsurgical hypocalcemia. Despite receiving those supplementations, hungry bone syndrome developed approximately 1 week after the parathyroidectomy, and her serum calcium and phosphate levels decreased to 8.1 and 1.1 mg/dL, respectively. As a result of the syndrome, intravenous calcium gluconate was given; the oral calcium carbonate dosage was raised to 4800 mg per day, while the alfacalcidol was increased to 3 μg per day to maintain a normal serum calcium level. The calcium and vitamin D supplements were gradually decreased over time, and the hungry bone syndrome had resolved by 10 months later.

After the serum calcium level had normalized (for our case, 10 days after the parathyroidectomy), closed reduction and internal fixation of the left proximal femoral shaft was performed. The orthopedic surgeon used a cephalomedullary nail (Zimmer Natural Nail; Zimmer Inc., Warsaw, Ind., USA). Other fractures were treated conservatively. The patient recovered well and was discharged 10 days after surgical fixation, with no postoperative complications.

**Fig. 3:**
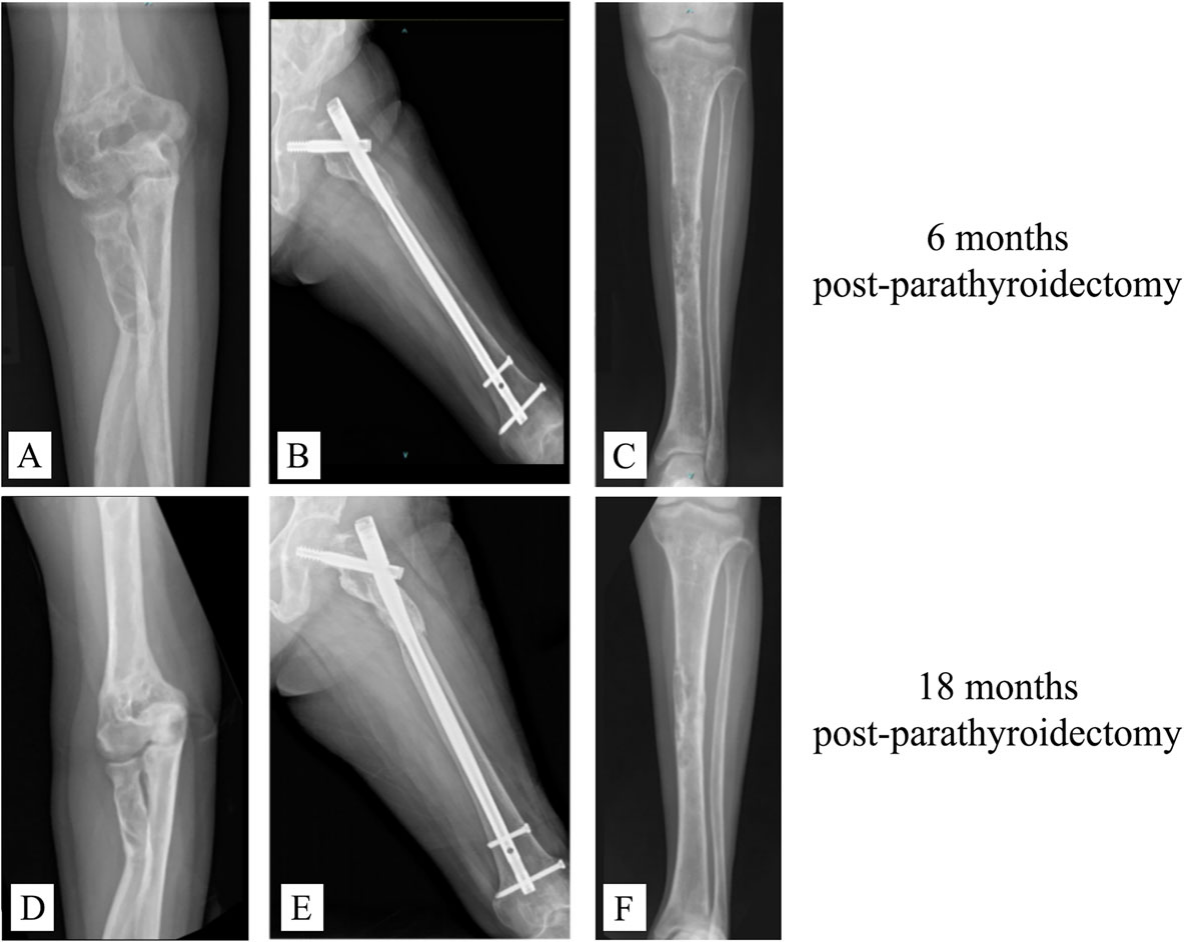
Plain radiographs of the patient at 6 months showing an increase in mineralization at (**a**) the right distal humerus, (**b**) the left femoral shaft, and (**c**) the left tibial shaft. Radiographs 18 months after surgery showing (**d**) union of the fracture at the right distal humerus and complete healing of the brown tumor; (**e**) union of the left proximal femoral shaft fracture after fixation with a long cephalomedullary nail; and (**f**) union of the left tibial shaft fracture after conservative treatment

Six months following the parathyroidectomy, the patient demonstrated a significant improvement in pain relief and level of ambulation. She switched from ambulation in a wheelchair to full weight bearing on her left leg. At the 1.5-year follow-up, plain radiographs of her extremities showed union of all fracture sites and complete healing of the brown tumors (Fig. 3). Although she was free of bone pain and able to walk without an assistive device, there was still some limping due to the shortened right femur.

## Discussion and conclusions

We report a case of PHPT that presented with multiple pathological fractures. A pathological fracture is one that occurs after a low-energy trauma to a pre-existing bone lesion [2]. Once a diagnosis of pathological fracture is made, metabolic bone diseases (such as PHPT and osteomalacia) and bone metastases should be investigated. The fracture rate in patients with PHPT has been reported to be approximately 15 per 1000 person years [3].

The skeletal manifestations of PHPT are bone pain and/or pathological fractures of the long bones; however, fractures are an uncommon initial presentation [4]. Osteitis fibrosa cystica is the classic radiographic feature of PHPT. It is characterized by a demineralized skeleton, a salt-and-pepper appearance of the skull, subperiosteal resorption of the phalanges, bone cysts, and brown tumors. Osteitis fibrosa cystica develops following prolonged exposure of bone to high serum PTH levels. Before the 1970s, the prevalence of osteitis fibrosa cystica was as high as 69% [5]. However, this condition has become increasingly uncommon in developed countries. This is because the introduction of routine serum calcium screening in the early 1970s led to early diagnosis of PHPT in asymptomatic cases [6]. In contrast, in countries such as Thailand where serum calcium level screening of healthy, asymptomatic individuals is not performed routinely, osteitis fibrosa cystica is still regularly reported. In Thailand, most osteitis fibrosa cystica patients (97.8%) present with symptomatic PHPT, and two-thirds have skeletal symptoms. However, the exact incidence of PHPT in the Thai population is still unknown [7]. With our patient, the presence of multiple fractures and radiographic evidence of notable bone changes attracted our attention and led us to perform further investigations to make a definite diagnosis. Although osteitis fibrosa cystica could be a presentation in cases of long-standing secondary HPT or tertiary HPT, our patient’s profile did not correspond to secondary HPT due to her high serum calcium level. As to tertiary HPT, that condition occurs in end-stage renal disease patients, which was not our patient setting.

Brown tumors are basically non-neoplastic. Their histopathology is characterized by multinucleated giant cells (osteoclasts), repetitive granulation tissue, vascular and proliferating fibrous tissue, and a brown hemosiderin deposition that imparts the color after which the lesions are named [8]. They commonly affect the jaws, skull, pelvis, clavicle, ribs, femurs, and spine as single or multiple lesions. Plain radiograph or computed tomography scan findings include lytic or multilocular cystic changes. Because of the similarity of the radiologic features of brown tumors with those of benign and malignant bone lesions (e.g., a cyst-like radiolucency), diagnosis can be difficult. There are some clues to differentiate brown tumors from other conditions. The severity of the bone pain arising from a brown tumor is milder than that from malignant bone lesions. Although symptoms of benign bone lesions (e.g., an aneurysmal bone cyst, simple bone cyst, or fibrous dysplasia) can mimic those of brown tumors, certain signs are only found with brown tumors, specifically, subperiosteal bone resorption on the skull, or a salt-and-pepper appearance and osteosclerosis of the upper and lower end plates of vertebrae (the rugger-jersey spine) [9]. The reported patient came to our hospital with reoccurring fractures at multiple sites. During the preceding 5 years, the individual fractures either had been associated with low-energy injuries or had had no history of trauma. A salt-and-pepper appearance and rugger-jersey spine were visible on plain radiographs.

Agarwal et al. retrospectively studied the biochemical markers of bone turnover, bone density, and radiographic recovery after parathyroidectomies in 51 patients who had PHPT and osteitis fibrosa cystica [10]. Remineralization occurred as early as 1 week after the procedures. In the case of the pelvis and spine, an improvement in osteopenia was observed within 3 weeks to 3 months, whereas recovery of the subperiosteal and cortical resorption of the skull and hands occurred after 6 to 9 months. The sites of the bone cysts, brown tumors, and fractures appeared abnormally dense on plain film X-ray within 3 months. In the present case, excision of the parathyroid adenoma resulted in an improved biochemical profile within a month of the surgery. Moreover, an increased bone density and healing of the brown tumors were noted in plain radiographs at the 6-month follow-up.

For orthopedic conditions, some studies have reported that orthopedic fixation was performed before a parathyroidectomy [6, 11], while other research has reported that the procedures were undertaken simultaneously [12]. In our case, the parathyroidectomy was performed first because the fractures could be temporarily immobilized externally. This was achieved through traction for the left femoral-shaft fracture and casting for the right-humeral and tibial-shaft fractures, before definite internal fixation. This approach is appropriate for patients with severe bone lesions and hypercalcemia. The surgery was delayed until the manifestations of hypercalcemia and intravascular volume were corrected, given that hypercalcemia may lead to adverse intraoperative events. However, if parathyroidectomy is chosen for the first operation, one should be aware of the possibility of hungry bone syndrome. This syndrome is characterized by rapid, profound, and prolonged hypocalcemia that is accompanied by hypophosphatemia and hypomagnesaemia. The hypocalcemia results from suppressed PTH after parathyroidectomies in patients with severe primary HPT and preoperative high bone turnover [13]. Until the hypocalcemia is resolved, definite fixation for the orthopedic condition should be delayed.

The left tibial fracture was treated conservatively with plaster of paris because of an acceptable alignment, whereas the distal humeral fracture was treated with a long arm U-slab due to severe fracture comminution. Operative treatment was chosen for the left proximal femoral fracture because of the benefits offered by an early postoperative-mobilization and rehabilitation protocol. The surgical management of fractures in patients with poor bone quality, in terms of the selection of the surgical technique and implant, is a major challenge. With our case, it was difficult to evaluate the quality of the reduction and fixation of the left proximal femur due to diffuse osteopenia and an unfixed tibial shaft fracture. We performed intramedullary nailing fixation for the femoral shaft fracture because we considered it to be superior to screw-plate fixation. This is because it is a less invasive procedure and offers a greater improvement in functional bearing, a higher rate of union, and better stability and mechanical solidity [14]. The nonoperative treatment in the presented case resulted in good union. It has been previously demonstrated that after a parathyroidectomy, bone returns to normal activity and fractures can be treated conservatively, provided they are non-displaced and uncomplicated [15].

In conclusion, in current practice, skeletal manifestations of PHPT are less common than in the past. A radiographic feature of PHPT is osteitis fibrosa cystica, which is characterized by a demineralized skeleton, a salt-and-pepper appearance of the skull, subperiosteal resorption of the phalanges, bone cysts, and brown tumors. If treated properly, PHPT is reversible. Although this condition has evolved to an asymptomatic disease for the majority of patients, its classic skeletal involvement should not be overlooked, particularly in young patients who present with multiple pathological fractures. In future, routine screening for PHPT in Thailand should be considered to avoid the full-blown manifestations of PHPT, like those found in the presented case.

**Table:** 

Learning points	
• Primary hyperparathyroidism is still a common endocrine and metabolic bone disorder, especially in the countries where the routine screening of serum calcium is not available.	
• Pathological fractures are not only caused by benign, malignant, or metastatic bone tumors; metabolic bone diseases should also be considered.	
• Bone metastasis and the radiological findings of osteitis fibrosa cystica can mimic each other. A salt-and-pepper appearance of the skull and a rugger-jersey spine appearance from bone survey may be used as clues to distinguish osteitis fibrosa cystica from other conditions.	
• The sequence of treatment should be discussed. o Parathyroidectomy before orthopedic fixation. ■ *Advantages*: normalized serum calcium; minimized risk of parathyroid crisis; improved intra-operative visualization of fluoroscopy; and stability of fixation (delayed fixation until improved bone mineralization is evident on radiograph). ■ *Disadvantages*: delayed fixation if hungry bone syndrome occurs; delayed early ambulation. o Orthopedic fixation before parathyroidectomy or simultaneous procedures. ■ *Advantages*: promotion of early ambulation; and decreased complications caused by immobilization. ■ *Disadvantages*: risk of parathyroid crisis; poor fluoroscopy visualization; and poor bone quality for implant fixation.	
• Fractures with an acceptable alignment that allows external immobilization to be applied can *effectively* heal after a parathyroidectomy.	
• A key to success is the use of a multidisciplinary approach involving an orthopedic surgeon, endocrinologist, radiologist, anesthesiologist, and otolaryngologist-head and neck surgeon.	

## Supplementary Information

**Additional file 1.** A plain radiograph of the previous fractures after internal fixations using plate and screws at (A) the right proximal humerus, (B) the supracondylar region of the left elbow, and (C) the right femoral shaft.

## Data Availability

The data that support the findings of this case report are available from the corresponding author on reasonable request.

## Abbreviations

ALP: Alkaline phosphatase
eGFR: Estimated glomerular filtration rate
PHPT: Primary hyperparathyroidism
PTH: Serum parathyroid hormone
