# Supplementary figures and images for: Osteitis Fibrosa Cystica and pathological fractures—the classic but neglected skeletal manifestation of primary hyperparathyroidism: a case report
Source: BMC Musculoskelet Disord. 2021 May 14;22:443. doi: 10.1186/s12891-021-04326-1 (PMC8122575; doi:10.1186/s12891-021-04326-1)

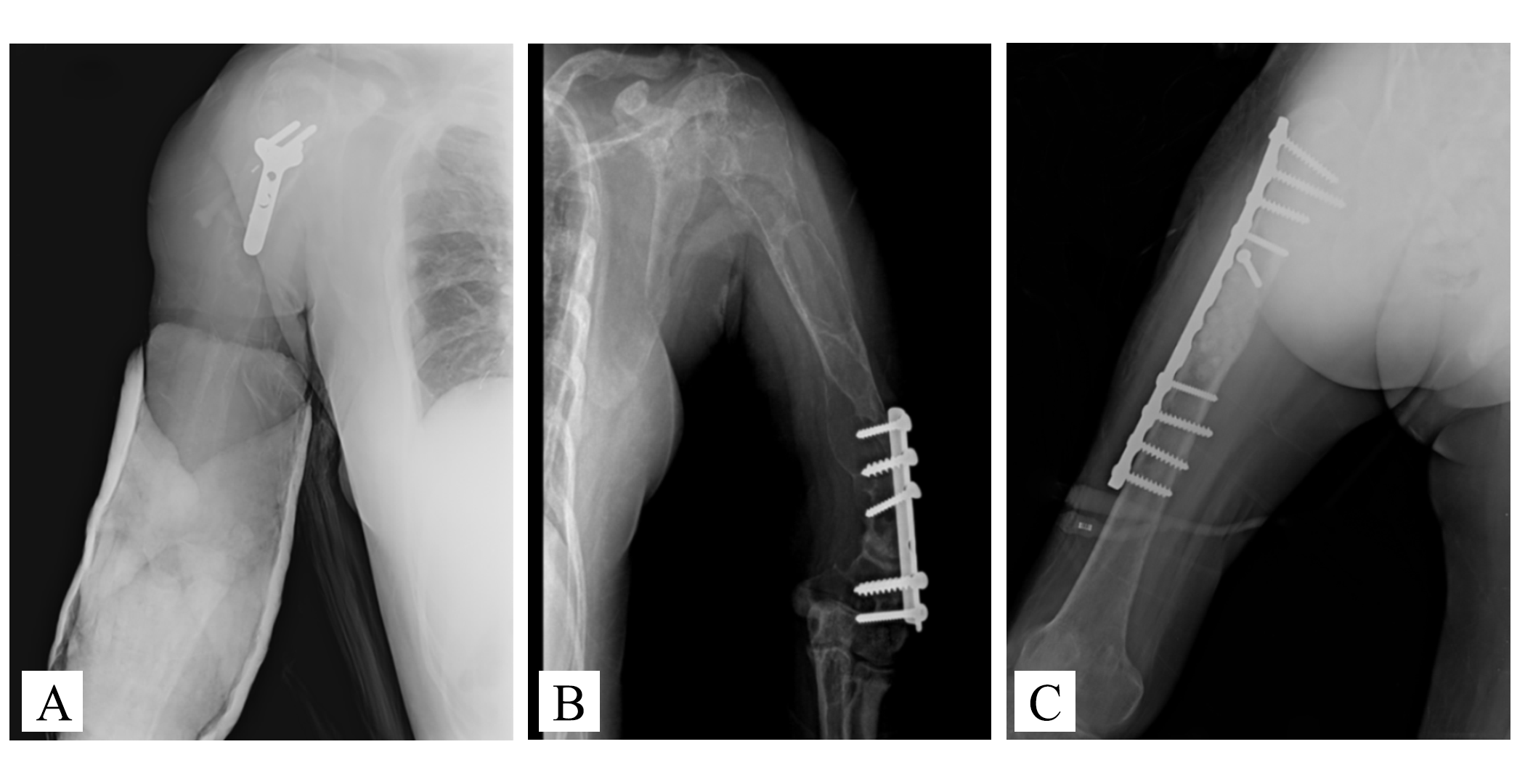

Supplement: Supplementary file 1 — Additional file 1. A plain radiograph of the previous fractures after internal fixations using plate and screws at (A) the right proximal humerus, (B) the supracondylar region of the left elbow, and (C) the right femoral shaft. [file 12891_2021_4326_MOESM1_ESM.tif]
